# Enzyme cocktail with hyperactive lipase through solid-state fermentation by the novel strain *Penicillium* sp. Y-21

**DOI:** 10.1038/s41598-023-41912-w

**Published:** 2023-09-04

**Authors:** Yang Cai, Guanghua Yang

**Affiliations:** 1 College of Food Science and Technology, Nanjing Agricultural University, Weigang #1, Nanjing, 210095 Jiangsu Province China; 2 College of Biological and Food Engineering, Changzhou University, Gehu Middle Road 21, Changzhou, 213164 Jiangsu Province China

**Keywords:** Microbiology, Applied microbiology, Fungi

## Abstract

Lipase is a kind of industrial enzyme preparation with various catalytic abilities and is widely used in food, energy, medicine and other fields. To increase lipase and enzyme cocktail activity through solid-state fermentation, the novel strain *Penicillium* sp. Y-21 was obtained through ethyl methanesulfonate (EMS) mutation from the novel strain Y, which was isolated from soils. Solid-state fermentation by strain Y-21 using agricultural byproducts was carried out in tray bioreactors. The optimum culture composition for enzyme cocktail fermentation was soybean meal 20 g, 3% (w/w) glucose, 1% (w/w) peptone, 5% (w/w) lard, 0.04% (w/w) CaCl_2_, 0.04% (w/w) FeCl_3_, 28 °C for 72 h. The enzyme cocktail produced by strain Y-21 is a kind of multienzyme complex, containing xylanase, glucanase, acidic protease, pectinase, cellulase and lipase, and their enzymatic activities (unit: U g^−1^) were 8000, 6000, 8000, 2000, 3000 and 120, respectively. During the fermentation process, the lipase coding genes *pel*, *pha*, and *p*12 were also studied and amplified from the RNA of *Penicillium* sp. Y-21 by RT-PCR. The results showed that the *pel* gene played an important role in enzyme production. Afterwards, an enzyme cocktail can be added to chicken feed as an additive, which improves animal growth and feed efficiency.

## Introduction

Lipases (EC3.1.1.3) are indispensable enzymes in industrial production. They act on triacylglycerol to release fatty acids and glycerol^1^, which can catalyze lipolysis, transesterification and ester synthesis^2,3^. At the same time, they have a wide range of substrate specificities, chemical selectivities, regioselectivities and stabilities in organic solvents, so they can be applied in the fields of organic synthesis, oil processing, food, medicine and other industries^4,5^. In nature, lipases are ubiquitous in seeds, animal tissues, and microorganisms^6^, where industrial lipases are mostly derived from microorganisms. At the beginning of 1834, Eber first detected lipase activity in rabbit pancreas. Lipases in microorganisms are rich in content, diverse in variety, widely distributed and superior to the adaptability of animals and plants. Therefore, microorganisms are currently the main method of industrial enzyme production^1,7–10^. In the microbial fermentation industry, the strain is a key factor that directly determines whether the product quality and yield are up to standard. Generally, the ability of the strains isolated from nature to accumulate products is very low, and it is impossible to achieve large-scale production requirements. Therefore, it is necessary to modify or improve the strains. There are three main methods of mutagenesis commonly used: physical, chemical, and compound mutagenesis^11^.

The exogenous enzyme cocktail from solid-phase fermentation could increase the nutrient digestibility and feed efficiency in livestock and poultry^12,13^. Feed substrate-specific enzymes cocktails are economically sustainability and have higher enzymatic hydrolysis of some specific chemical bonds present in the undigestible components of feed ingredients, such as plant materials. Protease, xylanase, glucanase, pectinase, cellulase, etc. are the most widely used feed enzymes. Plant feed contains a large amount of the nonstarch polysaccharide (NSP) fraction, which is poorly digested by livestock and poultry^14,15^. Exogenous enzyme cocktails, such as carbohydrases, can break specific chemical bonds in plant-based diets^13,15^. Protease and lipase can improve the digestibility of amino acids and dietary lipids, respectively^16,17^. Currently, multienzyme complexes contain lipase used for feed supplementation are seldom reported. Although microbial lipase is easy to find, it is very difficult to find a substrate-specific lipase-producing strain for solid-phase fermentation that can produce an enzyme cocktail containing acid protease, xylanase, glucanase, pectinase, cellulase and lipase.

In this study, we utilized low-cost crop waste soybean meal to reduce the production cost of lipase, and adopted solid-state fermentation to produce an enzyme cocktail containing lipase, which saves equipment investment and production operation costs compared with liquid fermentation and does not easily cause bacterial contamination during operation. At the same time, this experiment also provides a method for chemical mutagenesis of lipase-producing strains, purification and gene expression of lipase. Finally, the enzymatic characteristics show that the lipase has good conditions at pH 7.0 and 40 °C. The pH stability and temperature stability make this strain have good application prospects in feed biotechnology.

## Materials and methods

### Chemicals and microorganism

Ethyl methanesulfonate (EMS), DNA and protein Marker, 2 × Loading Buffer, SDS-PAGE gel preparation kit, TRIzol Lysate, M-MuLV First-Strand cDNA Synthesis Kit, and 2 × SG Fast qPCR Master Mix were purchased from Sangon Biotech (Shanghai); glucose, peptone, ammonium sulfate, olive oil and other chemical reagents were purchased from Sinopharm Chemical Reagent.

Wild type *Penicillium* sp. Y, was harvested from the soils as described previously^18^. The strain was stored at Changzhou University and revived and grown on slant media for further study. The spores of *Penicillium* sp. Y, were prepared by washing a 5-day incubation on plate agar with 0.85% sterilized NaCl solution. The spores were added to the sterilized solid fermentation medium to reach a final concentration of 10^6^ spores per gram of dry solid matter.

### EMS mutagenesis

The spores of *Penicillium* sp. Y were obtained by washing a 5-day incubation on plate agar. A certain amount of spore suspension and original EMS solution were used to prepare spore suspensionS containing 4% EMS (w/w)^19^. After mixing and shaking for 0–90 min, the control group received the same volume of saline as EMS. After mutagenesis, 2% Na_2_S_2_O_3_ of the same volume as EMS was added, and after standing for 10 min, the mixture was centrifuged at 3,000 rpm for 5 min and 1 mL of treatment solution was left. The treatment solution was diluted 100 times with sterile water and coated.

### Solid-state fermentation substrate composition

Wheat bran, soybean meal, rice bran, cottonseed meal and rapeseed meal were used as the main carbon and nitrogen sources. Then the total moisture content of the substrate was controlled at 60%. Optimization of fermentation considered substrate composition, additional carbon and nitrogen sources and their concentrations, different metal ions and concentrations, water content, inoculum and the effect of induced oil on enzyme cocktail production. The spores of *Penicillium* sp*.* Y-21 were inoculated into fermentation medium, and cultured at 28 °C for 72 h.

### Tray bioreactor design and configuration

**Figure 1:**
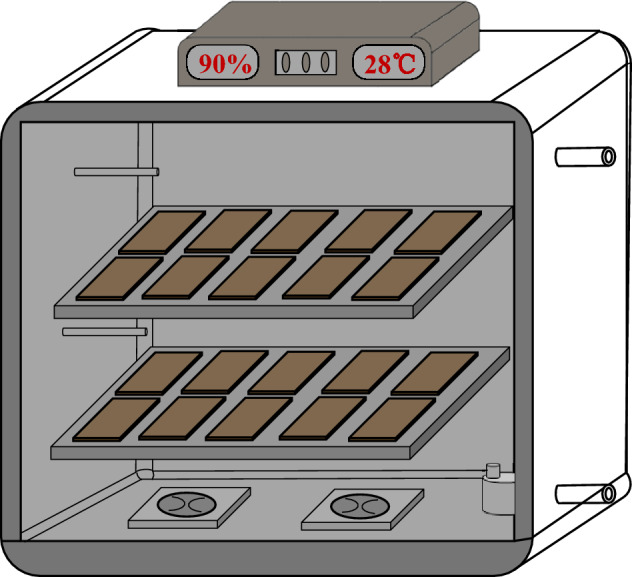
Diagrams of bench scale tray bioreactor.

The bioreactor (Fig. 1) was applied for the fermentation of cocktail lipase. The filtered air entered the chamber from the bottom, passed around the trays, and went out from the top. The bottom of the trays was perforated (2–3 mm) to allow the oxygen into the fermentation medium. The fermentation bed was approximately 1.5 cm deep to avoid any temperature gradient across the bed^20^. The temperature in the fermentation chamber was set at 28 °C, and the air moisture was approximately 70–80% to minimize water loss caused by evaporation through the bed. The trays were filled with 300 g of dry substrate, and samples were taken every 12 h for determination of lipase activity. The duration of the fermentation process was 72–96 h.

### Enzyme production and purification

The lipase was extracted using 0.1 M phosphate buffer (pH 7.2) as follows: 30 mL of phosphate buffer was added into the flask with 5 g of 72 h fermentation culture and placed in a shaker at 28 °C, and 200 rpm for 30 min. Then, the mixture was centrifuged at 5,000 rpm and 4 °C for 10 min. The supernatant was the crude lipase solution and kept at 4 °C for further analysis and purification. Lipase purification was performed according to previous methods^21–23^.

### Lipase-related gene expression

The RNA of the induced oil and the oil-free fermentation strain was extracted by using TRIzol lysate, and then cDNA was synthesized (see M-MuLV First-strand cDNA Synthesis Kit) using qRT-PCR to explore the relationship between the expression level of related genes and the enzyme activity in the lipase production process. Three pairs of primers were designed by Primer6 software to detect the dynamic changes in related genes in the presence of induced oil.

### Animal experimental procedures and measurements

A total of 9000 yellow broilers were collected and randomly divided into two group: control and treatment. Broiler chickens were randomly assigned into 2 experimental diets using a factorial arrangement with 2 enzyme cocktail supplemental levels (0 and 0.05%, w/w) in basal diets. The basal diet was formulated according to the Poultry Nutrient Requirements (Ministry of Agriculture of the People’s Republic of China, 2004). The birds were housed in a factory room and allowed ad libitum access to the diets and water. The room temperature was maintained at approximately 25 ± 2 °C. Feed intake was recorded daily to calculate the average daily feed intake (ADFI, g day^−1^). The weight of each group of broiler chickens was weighed at 1, 21, 22, and 50 d to calculate the average daily gain (ADG, g day^−1^). The feed conversion rate (FCR) was calculated with ADFI and ADG. The study was approved by Ethics Committee of Changzhou University, all methods were carried out in accordance with relevant guidelines and regulations. And all methods in this study are reported in accordance with ARRIVE guidelines.

### Analytical methods

The enzyme activity of lipase was determined by means of GB/T 23535-2009. The unit of lipase activity is defined as hydrolyzing 1 μmol of titratable fatty acid within 1 min under a certain temperature and pH (U g^−1^). The protein content was determined by the Bradford method using bovine serum albumin (BSA) as the standard protein^24^.

### Ethical approval

This article does not contain any research with human or animal subjects performed by any of the authors.

## Results and discussion

### Chemical mutation of strain 21

Based on the previous test results, 4% EMS solution was used for wild-type strain Y mutagenesis for 80 min to increase the lipase activity. Twenty three mutant strains were selected to determine the lipase activity (Table S1). Strain 21, with the highest lipase activity, was selected for further study, and the enzyme activity reached 73.33 U g^−1^. Compared with 48.67 U g^−1^ of the wild-type strain, the enzyme activity increased by 50.67%. Strain 21 was named *Penicillium.* sp. Y-21 according to 18S rRNA sequence results.

**Figure 2:**
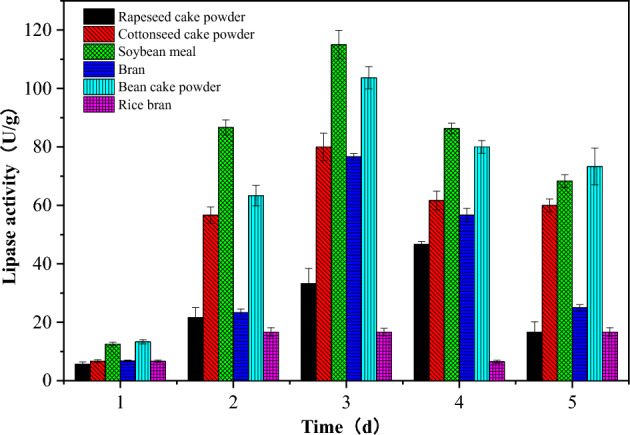
Fermentation growth curves of strains in different solid substrates.

Different substrates, such as wheat bran, soybean meal, rice bran, cottonseed meal and rapeseed meal, were selected as carbon and nitrogen sources for enzyme cocktail fermentation. The results showed that the maximum enzyme activity occurred in the soybean meal substrate by the mutant *Penicillium* sp.Y-21. The activity of lipase reached 115.60 U g^−1^ after 72 h fermentation at 28 °C (Fig. 2).

### Effect of inoculant and water content on lipase fermentation

**Figure 3:**
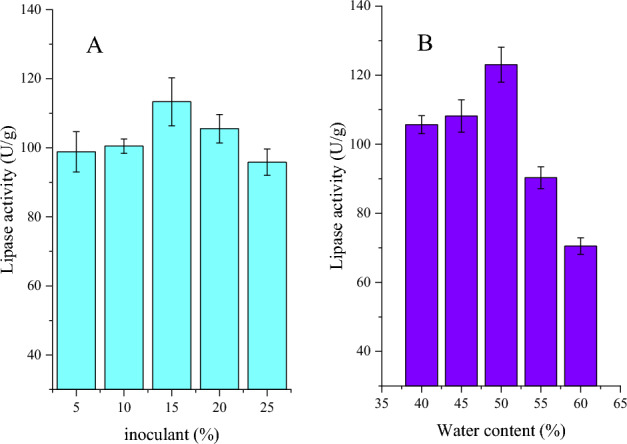
Effect of inoculant (**A**) and substrate moisture (**B**) on enzyme fermentation.

The effects of inoculation amount and moisture on enzyme production by strain 21 were investigated. The results are showed in Fig. 3. Strain 21 with 15% spore inoculant had the maximum enzyme fermentation and its activity reached 112.57 U g^−1^ in the substrate with 20 g soybean meal. Less inoculant could lead to longer fermentation times, and higher inoculants also affect enzyme production^25^. Substrate moisture also affected the enzyme fermentation and activity (Fig. 3B). Lipase activity was 123.04 U g^−1^ under the condition of 50% water content. The excessive amount of water added is not conducive to the ventilation and heat dissipation of the solid medium, which leads to lower enzyme activity.

### Effect of additional carbon/nitrogen source on solid state fermentation

**Figure 4:**
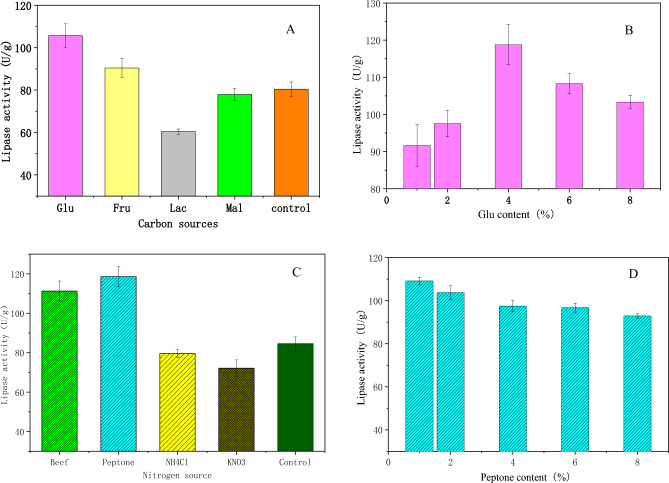
(**A**). Effect of additional carbon source and its content on enzyme production. (**B**). Effect of carbon source concentration on enzyme production by strains. (**C**). Effect of additional nitrogen source on enzyme production by strains. (**D**). Effect of nitrogen source concentration on enzyme production by strains.

Glucose, fructose, lactose and maltose were added to the fermentation medium as additional carbon sources, and the optimal carbon concentration was also studied. The results are shown in Fig. 4A,B. Additional glucose can improve enzyme production and activity. Lactose and maltose have an inhibitory effect on enzyme fermentation, and 4% glucose content is the optimal concentration for the enzyme production of the strain, and the enzyme activity reaches 115.67 U g^−1^ because glucose can be directly utilized as monosaccharide, which is beneficial to enzyme production^26,27^.

Beef extract, peptone, ammonium chloride and potassium nitrate were added to the culture medium as external nitrogen sources. Figure 4C and D show that additional peptone is favorable for enzyme production. Ammonium chloride, potassium nitrate and other inorganic nitrogen sources can decrease enzyme activity; 1% peptone is appropriate for enzyme production, and the lipase activity can reach 119.17 U g^−1^. Organic nitrogen peptone contains not only protein and amino acids but also a small amount of sugar and growth factors. Therefore, the growth effect of the strain was significantly improved.

### Effect of additional metal ions on solid state fermentation

**Figure 5:**
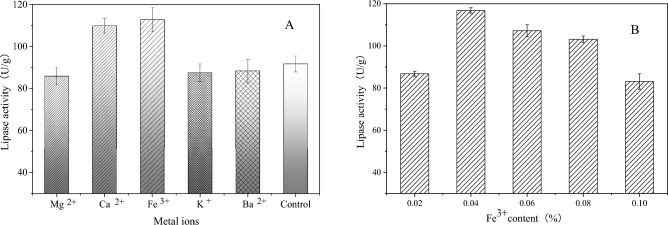
(**A**). Effect of metal ions on enzyme production by strains. (**B**). Effect of Ca^2+^/Fe^3+^ concentration on enzyme production by strains.

Mg^2+^, Ca^2+^, Fe^3+^, K^+^ and Ba^2+^ were added to the fermentation medium, and then optimum concentrations of metal ions of 0.02%, 0.04%, 0.06%, 0.08% and 0.1% were selected. The results showed that the addition of Fe^3+^ greatly enhanced the enzyme-producing ability of the strain, as shown in Fig. 5A,B.

### Effect of additional oil on solid-state fermentation

**Figure 6:**
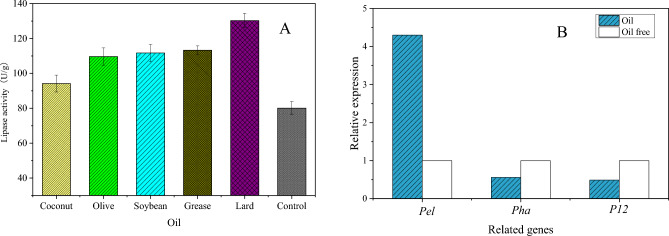
Effect of induced oil on lipase production.

Olive oil, coconut oil, soybean oil, grease and lard were added to the fermentation medium, and the results are shown in Fig. 6A. It can be seen from the figure that the strain is an oil-inducing strain, and the ability to produce lipase is greatly enhanced by adding oil. The lipase activity reached 120.33 U g^−1^ after adding lard. The reason may be that lard contains more polysaturated fatty acids, which can improve the enzyme production of the strain.

The qRT-PCR results showed that the peak value of the fusion curve was single, which indicated that the primers had high specificity and no nonspecific band amplification. According to the results of relative quantitative analysis in Fig. 6B, the gene expression of *pel* after adding induced oil was 4.30 times that without adding oil, and the gene expression of *pha* and *p12* were 0.56 and 0.49 times that without adding oil, as shown in Fig. 5B. The results showed that high expression of the *pel* gene could effectively improve the lipase production of the strain, while the *pha* and *p12* genes played a negative role in lipase production.

### Cocktail lipase fermentation and purification

Based on the optimum results shown above, a tray bioreactor was designed for lipase production. The ctivity of lipase, proteinase and xylanase were determined during fermentation and the results are shown in Table S3. Lipase and proteinase were generated simultaneously during the solid fermentation process, and their activity reached a maximum at 72 h of fermentation. Lipase and proteinase production were significantly higher in the tray bioreactor, and their production yields are 75 and 35% higher in the tray bioreactor than in the flask bioreactor. The results were in accordance with previous reports that filamentous fungi had better performance for enzyme production in static bed^28–32^.

**Figure 7:**
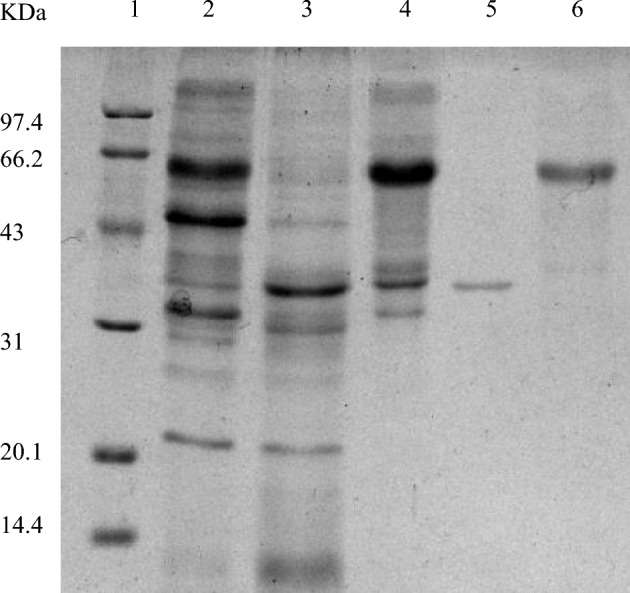
SDS-PAGE electrophoresis of enzyme cocktail, purified lipase and protease (1. Marker; 2. Enzyme cocktail; 3. 50% ammonium sulfate; 4. 80% ammonium sulfate; 5. Purified lipase; 6. Purified protease).

The lipase and proteinase were purified 63.24 and 10.33 times, respectively, through ammonium sulfate precipitation and chromatography. The recovery rates were 13.67% and 14.39%, and the specific activities were 1366.67 U mg^−1^ and 8540 U mg^−1^, respectively, as shown in Table S4. First, the protein was precipitated with ammonium sulfate at 50% saturation, and the target protein was purified by ion chromatography. The SDS-PAGE of the purified protein is shown in Fig. 7. The purified lipase is a single band with a molecular weight of 36.2 kDa (lipase) and 64.7 kDa (proteinase).

### Characterization of enzyme cocktail

The cocktail enzyme activities and characteristics were investigated. The results showed that the enzyme cocktail contained xylanase, glucanase, acidic protease, pectinase, cellulase and lipase, and their enzymatic activity units were 8000 ± 600 U g^−1^, 6000 ± 500 U g^−1^, 8000 ± 600 U g^−1^, 2000 ± 150 U g^−1^,3000 ± 200 U g^−1^ and 120 ± 50 U g^−1^, respectively. This enzyme cocktail includes relatively high xylanase, glucanase and protease activity but relatively low pectinase, cellulase and lipase activities, especially lipase activity, which was minimumal and only reached 120 U g^−1^.

Dietary supplementation of animal diets with exogenous enzymes has been suggested as a strategy to increase nutrient digestibility and improve feed efficiency. Generally, 0.1% of exogenous enzyme cocktail (w/w) is usually added to chicken and pig feed as an additive (DB33/T 459–2003). Excessive enzyme cocktail loading is recommended by the association to increase the efficiency of substrate digestibility. To understand the enzymatic hydrolysis effect, 9000 yellow broilers were collected to verify the substrate digestibility efficiency after adding 0.05% of cocktail enzyme. Nine thousand yellow broilers were divided into two groups: control and treatment. The average daily gain (ADG, g day^−1^), average daily feed intake (ADFI, g day^−1^) and feed conversion rate (FCR) were monitored and analyzed as shown in Table S5.

As shown in Table S5, ADG was 17.12 g day^−1^ and 33.15 g day^−1^ after adding the cocktail enzyme in days 1–21 and 32.57 in days 1–50, respectively, and ADG increase by 0.58 g day^−1^ in 50 d. In addition, ADFI and FCR decreased by 1.39 g day^−1^ and 0.05 in 50 d, respectively. Especially during days 22–50, the ADFI decreased by 2.34 g day^−1^, and the FCR decreased by 0.08, which showed that the addition of the cocktail to the chicken feed could significantly improve animal growth and increase feed efficiency in this study. The multienzyme complexes used in the experiments had combinations of 6 different enzymes and the enzymes xylanase, glucanase, acidic protease, pectinase, cellulase and lipase. An increasing number of studies in which enzymes are supplemented both individually and in combination are needed to determine the additive effect of enzyme supplementation in grow-finisher diets^13,33–36^. The enzyme cocktail in this study is a kind of multienzyme complex supplementation, that also depends on the ingredient content and most consistently improves growth and feed efficiency^33–36^.

## Conclusion

The results showed that the lipase activity of *Penicillium* was 125.33 U/g after mutagenesis by ethyl methylsulfonate. Through gene level detection, it was found that high expression of the *pel* gene could enhance the enzyme activity of the strain. After freeze-drying, the enzyme activity reached 330 U/g. and then purified by ammonium sulfate precipitation, anion chromatography and concentration. The recovery rate was 13.67%, and the purification multiple was 63.24 times. After SDS-PAGE electrophoresis, a single band was clearly identified, and the relative molecular weight of the lipase was 36.2 kDa. By studying the enzymatic properties of the lipase, we found that the optimum reaction temperature and pH of the lipase were 40 °C and 7.0, respectively. Therefore, *Penicillium* sp. Y-21 strain can be used as an important source of enzyme cocktail production containing xylanase, glucanase, acidic protease, pectinase, cellulase and lipase, and the enzyme cocktail could be used as an additive in feed to improve feed efficiency.

### Supplementary Information

Supplementary Tables.

## Data Availability

The datasets generated and/or analysed during the current study are not publicly available due to the industrial production of Jiangsu Youheng Biotechnology Co., Ltd, which supports the findings of this study. The datasets support the industrial fermentation conditions and were designed. However, they are available from the corresponding author on reasonable request.
