# Supplementary material for: Enzyme cocktail with hyperactive lipase through solid-state fermentation by the novel strain Penicillium sp. Y-21
Source: Sci Rep. 2023 Sep 4;13:14527. doi: 10.1038/s41598-023-41912-w (PMC10477218; doi:10.1038/s41598-023-41912-w)
Supplement: Supplementary file 1 — Supplementary Tables. [file 41598_2023_41912_MOESM1_ESM.docx]

**Supplementary Material**

**Table S1** Screening of mutagenized strains of ethyl methanesulfonate (EMS).

| No. | Enzyme activity（U/g） | Mortality rate（%） | No. | Enzyme activity（U/g） | Mortality rate（%） |
| --- | --- | --- | --- | --- | --- |
| #1 | 50 | 65.67% | #13 | 53.33 | 80.03% |
| #2 | 63.33 | 65.67% | #14 | 50 | 80.03% |
| #3 | 53.33 | 70.15% | #15 | 20 | 80.03% |
| #4 | 66.67 | 70.15% | #16 | 50 | 80.03% |
| #5 | 20 | 70.15% | #17 | 36.67 | 80.03% |
| #6 | 60 | 70.15% | #18 | 70 | 80.03% |
| #7 | 70 | 70.15% | #19 | 46.67 | 80.03% |
| #8 | 23.33 | 80.03% | #20 | 36.67 | 80.03% |
| #9 | 20 | 80.03% | #21 | 73.33 | 85.8% |
| #10 | 53.33 | 80.03% | #22 | 46.67 | 85.8% |
| #11 | 55 | 80.03% | #23 | 16.67 | 85.8% |
| #12 | 56.67 | 80.03% |  |  |  |

**Table S2** Sequence of primer pairs for qRT-PCR

| Gene | Primer sequences（5^，^-3^，^） |
| --- | --- |
| *Pel-F* | GAATTCGCAACTGCAGACGCTGCTGCCTT |
| *Pel-R* | GCGGCCGCTTATCAGCTCAGATAGCCACAACCAGCA |
| *Pha-F* | GATATGTTGTTCAACTACC |
| *Pha-R* | CTCAGCTCAGATAGCCACAAC |
| *P12-F* | CGCGGATCCACCATGTTGTTCAACTAC |
| *P12-R* | CCGGAATTCTCAGCTCAGATAGCCACAA |
| *β-actin-F* | GATGAGATTGGCATGGCTTT |
| *β-actin-R* | GTCACCTTCACCGTTCCAGT |

**Table S3 Cocktail enzyme activity**

| Kind of enzyme | Xylanase | Glucanase | Protease | Pectase | Cellulose | Lipase |
| --- | --- | --- | --- | --- | --- | --- |
| Acvitity (U/g) | 8500 | 5800 | 8000 | 1900 | 3000 | 120 |

**Table S4** Purification of lipase and proteinase

| steps |  | Activity (U) | Recovery rate（%） | Specific activity（U/mg） | Purification multiple |
| --- | --- | --- | --- | --- | --- |
| Crude enzyme | lipase | 600 | 100 | 21.61 | 1.00 |
|  | proteinase | 14832.53 | 100 | 826.32 | 1.00 |
| Salting out | lipase | 224 | 37.33 | 74.67 | 3.46 |
|  | proteinase | 10977.11 | 74 | 839.87 | 1.02 |
| chromatography | lipase | 82 | 13.67 | 1366.67 | 63.24 |
|  | proteinase | 2135 | 14.39 | 8540.00 | 10.33 |

**Table S5** ADG, ADFI and FCR of 9000 yellow broilers before and after adding exogenous cocktail enzyme

| **Items** | Control | Treatment | Control | Treatment | Control | Treatment |
| --- | --- | --- | --- | --- | --- | --- |
|  | 1-21 d | | 22-50 d | | 1-50 d | |
| ADG（g/d） | 17.05 | 17.12 | 44.52 | 45.10 | 32.57 | 33.15 |
| ADFI（g/d） | 27.95 | 27.29 | 97.07 | 94.73 | 67.04 | 65.65 |
| FCR | 1.64 | 1.60 | 2.18 | 2.10 | 2.03 | 1.98 |
